# The Effect of Intensive Statin Therapy on Symptomatic Intracranial Arterial Stenosis

**Published:** 2018-02

**Authors:** Peiyang ZHOU, Zhihua CAO, Pu WANG, Guangzhi LIU, Xuan YAO, Puqing WANG, Guang LI, Guibin ZHANG, Ping GAO

**Affiliations:** 1. Dept. of Neurology, Xiangyang No.1 People’s Hospital, Hubei University of Medicine, Xiangyang, China; 2. Dept. of Radiology, Xiangyang No.1 People’s Hospital, Hubei University of Medicine, Xiangyang, China

**Keywords:** Statin, Enhanced lipid-lowering, Intracranial atherosclerotic stenosis, CT perfusion imaging

## Abstract

**Background:**

The aim of this study was to observe the effect of intensive statin therapy on symptomatic intracranial arterial stenosis.

**Methods:**

overall, 120 patients with symptomatic intracranial arterial stenosis were admitted to the Xiangyang No.1 People’s Hospital, Hubei University of Medicine, Xiangyang, China from January 2010 to May 2013. They were randomly divided into three groups and were given different doses of atorvastatin orally for 1 year or more, and followed up for 12 months. The three groups were assessed for clinical end-point event rates and changes in cerebral blood flow value before and after treatment to assess the effectiveness of intensive statin therapy.

**Results:**

The incidence rates of end-point cerebrovascular events in the low-dose group (10 mg/d), the general-dose group (20 mg/d) and the intensive treatment group (40 mg/d) were 26.3%, 13.5% and 5.4% respectively during the 12-month follow-up after treatment. There was a significant difference between the low dose group and the intensive treatment group (*P*<0.05). The relative cerebral blood flow and relative cerebral blood volume of the three groups were significantly higher than those before treatment (*P*<0.05), and the relative time to peak for the intensive treatment group was shorter than that before treatment (*P*<0.001).

**Conclusion:**

Atorvastatin at 40 mg/d has a significant advantage compared with atorvastatin at 20 mg/d and 10 mg/d in reducing cerebrovascular events and improving cerebral blood flow.

## Introduction

Symptomatic intracranial atherosclerotic stenosis (sICAS) is one of the important mechanisms of ischemic stroke. Despite the best treatment regimens, patients with sICAS still have a high risk of stroke (1).

Statins are reductase inhibitors that delay the progression of atherosclerosis, stabilize atherosclerotic plaques, prevent or reverse atherosclerotic plaques and even reduce the degree of atherosclerosis (2, 3). The current clinical evaluation of statins in stroke patients is limited to extracranial arteries.

The purpose of this study was to investigate the role of atorvastatin in the progression of intracranial arterial stenosis by CT perfusion imaging (CTP).

## Methods and Materials

### Research subjects

This study was a prospective and observational study led by a single institute. One-hundred-twenty cases of ischemic cerebrovascular diseases were selected from patients admitted to the Xiangyang No.1 People’s Hospital, Hubei University of Medicine, Xiangyang, China from January 2010 to May 2013. Among them there were 82 males and 38 females aged 50–80 (63.44 ± 9.86) years.

The patients were randomly assigned into 3 groups, with 40 patients in group A (atorvastatin 10 mg/d), 40 patients in group B (atorvastatin 20 mg/d) and 40 patients in group C (atorvastatin 40 mg/d). Atorvastatin was manufactured by Pfizer Pharmaceuticals, Inc.(trade name Lipitor) and was administered orally to the patients for at least 12 months until completion of the second clinical visit. There were 21 patients with TIA and 99 patients with cerebral infarction. All patients were diagnosed with dyslipidemia with 75 cases of hypertension, 23 cases of diabetes and 49 cases of smoking history. The body mass index (BMI) was (23.41 ± 2.05) kg / m^2^, LDL- HDL-C (3.95 ± 0.21) mmol / L and high-sensitivity C-reactive protein (7.31 ± 0.65) mg /l. The stenosis degree of intracranial artery was 50% ∼ 99%.

There were 101 cases with moderate stenosis, 19 cases with severe stenosis. Among the cases, there were 87 cases with middle cerebral artery stenosis and 33 cases with basilar artery stenosis. The mean stenosis degree was (63.5 ± 14.76) %. The relative cerebral blood flow (rCBF) was 0.71 ± 0.09, while the relative cerebral blood volume (rCBV) was 0.80 ± 0.13. The relative time to peak (rTTP) was 1.29 ± 0.15. There was no significant difference among the three groups in the general data and the groups were comparable.

The study was approved by the Ethics Committee of Xiangyang No.1 People’s Hospital, Hubei University of Medicine and written informed consents were signed by the patients and/or guardians.

### Inclusion criteria

1) Diagnosis of acute ischemic attack or transient ischemic attack (TIA) according to the diagnosis and treatment guidelines of acute ischemic cerebrovascular disease in China published in 2010; 2) Diagnosis of large artery atherosclerosis according to the TOAST criteria. CT angiography (CTA) confirmed middle cerebral artery or basilar artery stenosis, and the degree of stenosis ≥ 50%; 3) The clinical diagnosis of TIA or cerebral infarction etiology was related to stenosis of the intracranial artery; 4) TC> 5.2 mmol / L, or LDL> 3.6 mmol / L, with normal liver function.

### Exclusion criteria

1) Cerebral embolism and intracranial hemorrhage within 6 weeks or hemorrhagic cerebral infarction; 2) > 50% of extracranial artery stenosis on the same side as ipsilateral intracranial artery stenosis; 3) Chronic destructive diseases, significant heart and liver and other important organ failure and mental illness; 4) Patients with strokes caused by non-atherosclerotic vascular stenosis, including vasculitis, vasospasm, moyamoya disease and so on; 5) Patients allergic to statin, CTA agent, and drugs that increase the incidence of rhabdomyolysis when taken with statin drugs and other drugs that affect blood lipid levels; 6) Patients who could take care of themselves after cerebral strokes and who refused to participate in follow-ups after being discharged.

### Selection Evaluation

Two neurologists titled associate chief physician or above were chosen to select patients according to the criteria of inclusion and exclusion to assess whether the patient could be included in the study.

### Other medical treatment

All subjects were treated with a double-antibiotic therapy (aspirin tablets 0.1 g/d + clopidogrel 75 mg/d) when diagnosed with acute cerebral infarction or within 1 month after the onset of TIA. Aspirin tablets 100mg/d were still administered after 1 month. Drug was administered to control the blood pressure and blood sugar in patients with hypertension and diabetes.

### Clinical follow-up

Periodic follow-up, clinical follow-up, and clinical end points were assessed by full-time neurologists. The cerebrovascular end point events recorded included cerebral infarction and TIA. CTP was performed 1 week after the end of cerebrovascular event and 12 months after the administration of statin drug. Some patients underwent CTA examination at the same time.

### CTP Examination

Examination methods: GE's 64-layer Light speed VCT scanner was used. Scanning parameters: 120 Kv, 300 ∼ 350 mA, pitch 0.984, layer thickness 0.625 mm. First, 30 ∼ 40 ml of iohexol (350 mgI / ml) was injected through the elbow vein and 20 ml normal saline was infused at a rate of 4.0 ml/s. The dynamic perfusion scanning was performed after 5 seconds. The images obtained were transferred to AW4.3 standalone workstation and analyzed by Perfusion 2 software, generating pseudo-color function charts of perfusion parameters such as the cerebral blood flow (CBF), cerebral blood volume (CBV) and time to peak (TTP).

### Image analysis

All patients underwent CTP examination to have CBF, CBV, TTP pseudo-color images and gray-scale map obtained. The images were read by two senior radiologists. Region of interest associated with blood vessels with lesions were selected on the gray-scale map. Using the center line as the mirror, a mirror image of the region of interest was set on the opposite side. CBF, CBV and TTP were measured at four points of interest, and the values on the lesion side were divided by the corresponding values on the control side. The local relative values were therefore obtained, which include the relative cerebral blood flow (rCBF), relative cerebral blood volume (rCBV) and relative time to peak (rTTP). The average values at the four points were used to analyze the changes of cerebral blood perfusion before and after treatment.

### Statistical analysis

SPSS 17.0 (Chicago, IL, USA) statistical software package was used for statistical analysis of data. Quantitative data were expressed in mean ± standard deviation (x̅+S). Comparison within groups was conducted using the paired t test; comparison between groups was conducted using the independent samples t test; comparison among multiple groups was conducted with the use of variance analysis. Qualitative data were expressed using the composition ratio and percentage. Comparison between groups was conducted using the chi-square test (Fisher exact probability method). *p*<0.05 was considered statistically significant.

## Results

### Clinical follow-up

All 120 patients were followed up after 1 month of drug administration. Twelve months after the administration of drug, a total of 112 patients completed the treatment. In group A, 2 patients failed to continue because contact was lost with one patient and the other patient failed to adhere to the medication. In group B, 3 patients failed to continue because contact was lost with 2 patients and 1 patient failed to adhere to the medication. In group C, 3 patients failed to continue because contact was lost with one patient, one had ALT/AST three times more than the normal value, and one failed to adhere to the medication.

### Cerebrovascular end points

There was no deceased case in the three treatment groups during follow-ups. There was no significant difference in the incidence of end-point cerebrovascular events between group A, B and C at the 1-month follow-up. However, the incidence of end-point events in group C was significantly lower than that in group A at 12 months (*P*<0.05). There was no significant difference in the incidence of end-point cerebrovascular events between group A and B and between group B and C (Table 1).

**Table 1:** The incidence of end-point events during follow-ups at 1 month and 12 months (% cases)

***Group***	***TIA (n, %)***		***Cerebral infarction (n, %)***		***Total (n, %)***	
	At 1 month	At 12 months	At 1 month	At 12 months	At 1 month	At 12 months
Group A	2 (5)	6 (15.8)	2 (5)	4 (10.5)	4 (10)	10 (26.3)
Group B	1 (2.5)	3 (8.1)	1 (2.5)	2 (5.4)	2 (5)	5 (13.5)
Group C	1 (2.5)	1 (2.7)	1 (2.5)	1 (2.7)	2 (5)	2 (5.4) *
*P*					0.585	0.039

* Comparison between group A and C: *P*=0.014 < 0.05

### Comparison of CTP parameters before and after treatment

**Fig. 1:**
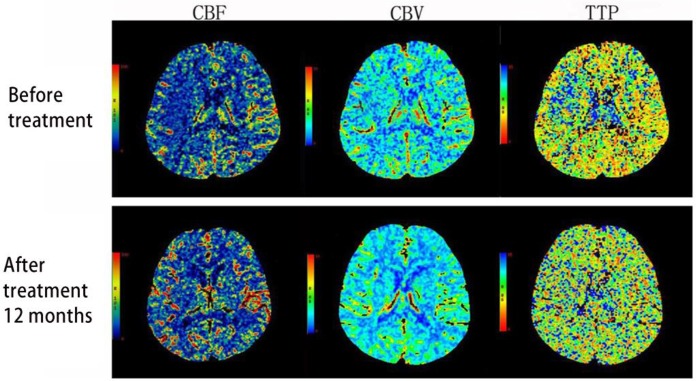
The CTP pseudo-color comparison of group C before and after the treatment: 12 months after the treatment, an increase in rCBF and rCBV and a decrease in rTTP were observed in the blood supply region of the left middle cerebral artery

Compared with before treatment, the rCBF and rCBV of group A, B and C 12 months after the treatment were significantly increased (*P<*0.001). The rTTP of group A and B showed no significant change while that of group C was significantly higher than before (*P*<0.05). Compared with group A and B, rCBF and rCBV of group C increased significantly while rTTP decreased significantly (*P*<0.001, Table 2, Fig. 1).

**Table 2:** Comparison of CTP parameters before and after treatment (X̅±S)

	***rCBF***		***rCBV***		***rTTP***	
	Before tx	12 mo after tx	Before tx	12 mo after tx	Before tx	12 mo after tx
Group A	0.72±0.06	0.79±0.08^a^	0.80±0.12	0.85±0.08^a^	1.30±0.11	1.34±0.08
Group B	0.71±0.07	0.81±0.08^a^	0.80±0.15	0.86±0.08^a^	1.31±0.12	1.35±0.09
Group C	0.71±0.11	0.86±0.08^^a^^b^^c^^	0.78±0.13	0.89±0.06 ^^a^^b^^c^^	1.25±0.15	1.22±0.07 ^^a^^b^^c^^

Note : Compared to before treatment,

a *P*<0.05; compared to group A,

b *P*<0.05; compared to group B,

c *P*<0.05

## Discussion

SICAS refers to the intracranial arterial stenosis caused by atherosclerosis and cerebral infarction or TIA occurred in the narrow arterial blood supply region. SICAS is a risk factor for recurrent cerebral infarction and TIA. Its pathogenesis is related to instability of stenotic plaques leading to thrombosis or exfoliation, distal arterial stenotic hemodynamics and stenotic perforator vessel occlusion (4, 5). Despite the best treatment regimens, the risk of stroke in patients with sICAS is still very high. SICAS often progresses, which is an important cause of the occurrence of vascular events or death. Therefore, the choice of treatment for safe, reasonable and effective secondary prevention has a very important clinical implication.

Statins in addition to a lipid-regulating effect, inhibit cell proliferation in smooth muscles and promote apoptosis, inhibiting inflammatory responses. Statins can also improve vascular endothelial cell function, reduce lipid deposition in the vascular endothelium, reduce the formation of foam cells and inhibit the activity and aggregation of platelets to stable plaques and reduce the volume of atherosclerotic plaques (6). Studies of intravascular ultrasound evaluation of rosuvastatin on coronary atherosclerotic burden confirmed for the first time that intensive statin (atorvastatin 80 mg/d) treatment can reverse the coronary atherosclerotic plaques, reducing vascular stenosis (7).

There was no clinical death case reported in this study. At the 1-month follow-up, the incidence of cerebrovascular end points in group A, B and C was 10.0%, 5.0% and 5.0%, respectively, which was similar to the one mentioned in the literature. At the 12-month follow-up, the incidence of cerebrovascular end points in group A, B and C was 26.3%, 13.5% and 5.4%, respectively. Compared to group A, group C has an incidence rate that is significantly lower. The results suggest that group C can significantly lower the incidence of cerebrovascular end-point events compared to group A. However, when group A and B or group B and C are compared, there was no significant difference. The reason might be that the follow-up was relatively short. With the extension of follow-up time, it may further reflect the advantages of intensive statin therapy in the reduction of cerebrovascular end points. In group C, the incidence of end-point events at the 1-year follow-up was lower than that reported in the literature (12.2%). However, considering that the level of intracranial arterial stenosis in the study group was 50% to 99% with an average vessel stenosis rate at (63.5 ± 14.76) %, which was lower than the level reported in the literature (70% ∼ 99%), the results cannot be statistically compared.

Acute ischemic cerebrovascular disease is ultimately caused by low perfusion leading to ischemic brain changes, while atherosclerosis and cerebral artery stenosis or occlusion are the direct causes. CTP is the functional imaging that reflects the cerebral hemodynamics, which shows abnormal blood perfusion in the brain and evaluates the status of cerebral ischemia. There are still different views regarding the sensitivity of CTP parameters to ischemia. CBF is the best indicator that shows the size and location of infarction (8). Some studies have shown that TTP is highly sensitive to cerebral ischemia, suggesting that TTP prolongation is related to the collateral circulation or slow blood flow and can be used to evaluate the collateral circulation (9). In addition, the high specificity of CBV diagnosis of infarction is reported (10). This study shows that, compared with time before treatment, the rCBF, rCBV of group C were significantly increased and the rTTP was significantly shortened 12 months after the treatment. Although no improvement in intracranial arterial stenosis was observed in group A and B, the cerebrovascular of the two groups may have different degrees of collateral circulation compensation, which would explain why rTTP was prolonged. Group C significantly improved the degree of stenosis in intracranial artery, lowered the average rate of arterial stenosis. Therefore, even without the collateral circulation compensation, the rTTP value is still shortened.

For patients with sICAS, atorvastatin therapy that lasts one year may delay the progression of atherosclerosis, improve cerebral blood perfusion, and reduce the incidence of clinical end-point cerebrovascular events. Radiology provides a more direct observation of how statins reverse plaques, improve intracranial artery stenosis and improve cerebral blood perfusion. However, this effect cannot be completely explained by its lipid-lowering function. It is also related to the multifunctionality of statins, such as inhibition of plaque neovascularization, reduction in the activity of metalloproteinases, anti-inflammatory effect that raises the nitric oxide bioavailability, repair of damaged blood vessel endothelium and its antithrombotic, fibrinolytic and neuroprotective effects (11).

## Conclusion

Since this study was initiated by a single institute, it was limited by the low number of samples and short observation time period, which may affect the results. In future studies, joint research can be done by collaborating with other institutes to expand the sample size and extend the observation and follow-up time to further validate the role of statins in sICAS.

## References

[1] Gorelick PB Wong KS Bae HJ Pandey DK (2008). Large artery intracranial occlusive disease: A large worldwide burden but a relatively neglected frontier. Stroke, 39: 2396–2399. 18535283 10.1161/STROKEAHA.107.505776

[2] Ní Chróinín D Asplund K Åsberg S (2013). Statin therapy and outcome after ischemic stroke: Systematic review and meta-analysis of observational studies and randomized trials. Stroke, 44: 448–456. 23287777 10.1161/STROKEAHA.112.668277

[3] Aboa-Eboulé C Binquet C Jacquin A Hervieu M Bonithon-Kopp C Durier J Giroud M Béjot Y (2012). Effect of previous statin therapy on severity and outcome in ischemic stroke patients: a population-based study. J Neurol, 260(1):30–7. 22729388 10.1007/s00415-012-6580-9

[4] Liebeskind DS Cotsonis GA Saver JL (2011). Collaterals dramatically alter stroke risk in intracranial atherosclerosis. Ann Neurol, 69: 963–974. 21437932 10.1002/ana.22354 PMC3117968

[5] Lau AYL Wong EHC Wong A Mok VCT Leung TW Wong KSL (2012). Significance of good collateral compensation in symptomatic intracranial atherosclerosis. Cerebrovasc Dis, 33: 517–524. 22538868 10.1159/000337332

[6] Amarenco P Bogousslavsky J Callahan A 3rd (2006). High-dose atorvastatin after stroke or transient ischemic attack. N Engl J Med, 355: 549–559. 16899775 10.1056/NEJMoa061894

[7] Nissen SE Nicholls SJ Sipahi I (2006). Effect of Very High-Intensity Statin Therapy on Regression of Coronary Atherosclerosis: the ASTEROID trial. JAMA, 295: 1556–65. 16533939 10.1001/jama.295.13.jpc60002

[8] Nabavi DG Cenic A Henderson S Gelb AW Lee TY (2001). Perfusion mapping using computed tomography allows accurate prediction of cerebral infarction in experimental brain ischemia. Stroke, 32: 175–183. 11136934 10.1161/01.str.32.1.175

[9] van Seeters T Biessels GJ Kappelle LJ (2016). Dutch acute stroke study (DUST) investigators: CT angiography and CT perfusion improve prediction of infarct volume in patients with anterior circulation stroke. Neuroradiology, 58:327–337. 26767380 10.1007/s00234-015-1636-z PMC4819789

[10] Castilla Guerra L del Carmen Fernández Moreno M López Chozas JM Jiménez Hernández MD (2008). Statins in stroke prevention: What an internist should know. Eur J Intern Med, 19: 8–14. 18206595 10.1016/j.ejim.2007.05.010

[11] Manocha D Bansal N Gumaste P Brangman S (2013). Safety profile of high-dose statin therapy in geriatric patients with stroke. South Med J, 106: 658–664. 24305522 10.1097/SMJ.0000000000000024

